# Validation of the kidney failure risk equation and its impact on referral strategies for chronic kidney disease: protocol for a retrospective cohort study using national claims and laboratory data in Thailand

**DOI:** 10.1136/bmjopen-2025-114382

**Published:** 2026-05-15

**Authors:** Jeerath Phannajit, Chirod Narkpaichit, Chaisiri Angkurawaranon, Chanchanok Aramrat, Faye Cleary, Rupert W Major, Warangkana Pichaiwong, Sirirat Anutrakulchai, Kearkiat Praditpornsilpa, Hugo C Turner, Dorothea Nitsch

**Affiliations:** 1 MRC Centre for Global Infectious Disease Analysis, School of Public Health, Imperial College London, London, UK; 2 Division of Clinical Epidemiology, Department of Medicine, King Chulalongkorn Memorial Hospital, Thai Red Cross Society, Bangkok, Thailand; 3 Division of Nephrology, Department of Medicine, Faculty of Medicine, Chulalongkorn University, Bangkok, Thailand; 4 Center of Excellence for Metabolic Bone Disease in CKD patients, Faculty of Medicine, Chulalongkorn University, Bangkok, Thailand; 5 National Health Security Office, Bangkok, Thailand; 6 Faculty of Medicine, Chiang Mai University, Chiang Mai, Thailand; 7 Medical Research Council Integrative Epidemiology Unit, Population Health Sciences, Bristol Medical School, University of Bristol, Bristol, UK; 8 School of Medical Sciences – Public Health and Epidemiology Division (SAPPHIRE Group), University of Leicester College of Life Sciences, Leicester, UK; 9 University Hospitals of Leicester NHS Trust, Leicester, England, UK; 10 Leicester, Leicestershire and Rutland Integrated Care Board, Leicester, UK; 11 Nephrology Division, Department of Medicine, Rajavithi Hospital, College of Medicine, Rangsit University, Bangkok, Thailand; 12 Faculty of Medicine, Khon Kaen University, Khon Kaen, Thailand; 13 Department of Non-Communicable Disease Epidemiology, Faculty of Epidemiology and Population Health, London School of Hygiene and Tropical Medicine, London, UK; 14 UK Renal Registry, UK Kidney Association, Bristol, England, UK

**Keywords:** Chronic renal failure, Health policy, Clinical Decision-Making, Risk Assessment, Health economics

## Abstract

**Abstract:**

**Introduction:**

Chronic kidney disease (CKD) is highly prevalent in Thailand and imposes a growing burden on the health system, driven by limited nephrology capacity and high rates of unplanned dialysis. The kidney failure risk equation (KFRE) estimates the risk of progression to kidney failure (KF) on age, sex, estimated glomerular filtration rate (eGFR) and urine albumin-to-creatinine ratio. This study aims to validate and, if required, recalibrate the four-variable KFRE for the Thai population and to assess the potential impact of KFRE-guided referral strategies on clinical care and health system performance.

**Methods and analysis:**

We will conduct a retrospective cohort study using linked, de-identified national health databases covering approximately 70% of the Thai population. Adult patients with CKD stages 3–5 will be included. KFRE performance will be evaluated at 2 and 5 years for discrimination and calibration. If miscalibration is identified, the model will be recalibrated using Cox-based methods. Simulations (1000 iterations) indicated that approximately 920 KF events by 5 years would be required to achieve the target standard errors for the calibration slope. A subsequent impact analysis will compare KFRE-guided referral with current Thai CKD guideline criteria and real-world practice using a decision-tree and Markov modelling framework.

**Ethics and dissemination:**

Ethical approval was obtained from the Ethics Committee of the Institute for the Development of Human Research Protections, Thailand (COA No. IHRP2025110), Imperial College London and the London School of Hygiene and Tropical Medicine. The requirement for informed consent was waived due to the use of anonymised secondary data. Findings will be disseminated through peer-reviewed publications, conferences and policy briefs to supplement evidence-based referral strategies and health system planning.

**STRENGTHS AND LIMITATIONS OF THIS STUDY**

- Large linked national claims and laboratory databases enable analysis in a real-world population representing approximately 70% of the Thai population.
- Use of routinely collected retrospective data may introduce misclassification, incomplete coding and missing laboratory or treatment dates.
- Potential selection bias arising from missing albuminuria data will be explored through prespecified sensitivity analyses, including multiple imputation.
- Kidney failure is defined primarily by sustained kidney replacement therapy initiation or transplantation, a health-system-dependent endpoint that may vary with access, service capacity and policy over time.
- Restriction to patients covered by the Universal Coverage Scheme may limit generalisability to populations insured under other schemes in Thailand.

## Introduction and rationale

 Chronic kidney disease (CKD), defined as reduced kidney function or structural abnormalities persisting for a minimum of 3 months, affects more than 700 million people worldwide.^1^,^3^ It is detected and monitored using the estimated glomerular filtration rate (eGFR) to assess kidney function, and albuminuria, defined as the presence of albumin in urine and commonly measured as the urine albumin-to-creatinine ratio (uACR), which reflects structural damage. Kidney failure (KF), or end-stage kidney disease, represents the most advanced stage of CKD, in which patients require kidney replacement therapy (KRT)—either dialysis or kidney transplantation, to sustain life. KRT is expensive and places a significant burden on healthcare systems, especially in low- and middle-income countries. In Thailand, the overall CKD prevalence is estimated at 16.7–17.5%, or approximately 12 million people.^4 5^ Over 1 00 000 patients are currently receiving KRT under government funding, which accounts for more than 10% of the national health expenditure and continues to rise^6^ . Although Thailand has about 12.9 nephrologists per million population (PMP)—slightly above the average of 10.1 PMP for upper-middle-income countries^5^—this still equates to only one nephrologist for every 12 000 people with CKD, highlighting the imbalance between disease burden and specialist capacity.

With limited nephrology resources, early identification of patients at high risk of progression to KF is essential. The risk varies substantially between individuals,^7^ and delays in referral contribute to a high rate of unplanned dialysis initiation during emergency admissions. In Thailand, the rate of unplanned dialysis initiation has increased from 40% to 60%, a trend linked to increased mortality.^6 8^ Accurate prediction of KF can facilitate earlier referral of those high-risk individuals to nephrology care, support shared decision-making and enable timely planning for dialysis modality, vascular access placement and pre-emptive transplantation.^7^ These measures improve patient outcomes and support more efficient allocation of nephrology resources to those most in need.

The 2024 Kidney Disease Improving Global Outcomes (KDIGO) Clinical Practice Guideline recommends using externally validated risk equations to estimate the probability of KF in people with CKD.^9^ Among these, the kidney failure risk equation (KFRE)^10 11^ offers a validated tool, using variables including age, sex, eGFR and uACR, to predict 2- and 5- year risks of progression to KF, enabling referral decisions based on predicted risk rather than arbitrary clinical thresholds.^12^ The equation has demonstrated strong predictive performance and has been validated in diverse populations globally. However, its performance varies across ethnic, geographic and healthcare context due to differences in CKD prevalence, progression patterns and healthcare practices.^13^,^15^

In Thailand, referral decisions to nephrologists are currently based on fixed criteria such as an eGFR below 30 mL/min/1.73 m², rapid eGFR decline, significant albuminuria or the presence of complications, and rely mainly on physician judgement without a standardised, risk-based tool^16^ . This highlights the need for local validation of the KFRE to enable precise prioritisation of high-risk patients, optimise the utilisation of limited resources and inform future guideline and policy development. This study aims to externally validate and, where necessary, recalibrate the KFRE in Thai patients with CKD stages 3–5 using the national health database. In addition, this study will project the potential health system impacts and economic implications of adopting a KFRE-guided referral strategy in the Thai health system.

## Objectives

### Primary objective

To externally validate the performance of the four-variable KFRE and assess the need for recalibration in predicting the 2- and 5-year risk of KRT among the Thai CKD population.

### Secondary objectives

1. To describe the current burden of CKD in Thailand and characterise existing referral practices.
2. To assess the robustness and generalisability of the KFRE across key subgroups, including CKD stage, diabetes status, sex, age group and albuminuria category, and across alternative eGFR equations and outcome definitions.
3. To evaluate the potential impact of implementing the KFRE in the referral system compared with current referral criteria.

## Methods and analysis

### Study population and eligibility criteria

We will include all adult patients (aged 18 years or older) with CKD stage 3–5, defined by at least two eGFR measurements below 60 mL/min/1.73 m² obtained ≥90 days apart, between 1 January 2017 and 31 January 2025. To avoid survival and observation bias, the index date (time zero for baseline risk calculation and the start of follow-up) will be defined as the date of the uACR (or uPCR) measurement, following the established methodology used in previous primary care KFRE validations.^13^ If uACR is unavailable, a urine protein-to-creatinine ratio (uPCR) within the same period will be used and converted to uACR using a validated equation.^17^ For the calculation of the KFRE risk score, the eGFR measurement recorded nearest in time to this index date will be utilised as the baseline eGFR. Patients will be excluded if they have evidence of chronic dialysis, prior kidney transplantation, documented palliative care or the occurrence of death or KRT initiation within the qualifying period. All participants will be followed for outcomes until 1 September 2025. A complete list of inclusion and exclusion criteria is provided in online supplemental appendix 1.

### Data sources and population coverage

The analysis will use retrospective de-identified patient-level data from four linked national databases (table 1). Cohort entry relies on laboratory data from the Health Data Centre (HDC), maintained by the Ministry of Public Health (MoPH). Outcomes and covariates will be obtained from the National Health Security Office (NHSO) databases: the Chronic Kidney Disease–Disease Management Information System (CKD-DMIS), which registers patients reimbursed for dialysis and transplantation; the electronic claims database (e-claim), which contains outpatient and inpatient visits, diagnoses, procedures and cost data; and the NHSO death registry, derived from linkage with the national Civil Registration Database maintained by the Ministry of Interior. All databases are linked using a de-identified, encrypted patient identifier. Data were collected from 1 January 2017 to 31 January 2025 and stored on secure NHSO servers.

**Table 1:** Summary of data sources

Data source	Description/coverage	Variable extracted
**1.Thai HDC—Laboratory data**	Contains all laboratory records from HDC-participating hospitals, including all MoPH-affiliated hospitals and participating sites outside the MoPH, from 2017 to 2025.	- Age, sex, hospital site and health insurance scheme (at the time of test) - Laboratory results including serum creatinine, uACR and uPCR.
**2.CKD-DMIS**—kidney replacement therapy registration	Registry of patients with KF reimbursed under the UCS managed by the NHSO covering all KRT modalities (haemodialysis, peritoneal dialysis and kidney transplantation) from 2012 to 2025.	Date of dialysis initiation or kidney transplantation Mode of KRT Vascular access (for haemodialysis) Temporary haemodialysis use (for peritoneal dialysis patients) Dates of individual haemodialysis sessions and peritoneal dialysis fluid reimbursement
**3.Electronic claim database** (e-claim)	Records of outpatient visits and inpatient admissions under the UCS including dates of visits, hospital site, diagnostic codes (ICD-10), procedural codes (ICD-9) and costs.	Diagnostic codes (ICD-10) and first recorded date of diagnosis for diabetes mellitus, hypertension, heart failure, coronary heart disease, atrial fibrillation, stroke, peripheral arterial disease, chronic obstructive pulmonary disease and acute kidney injury
**4.NHSO’s Death registry**	Records of patient deaths derived from linkage with the national Civil Registration Database maintained by the Ministry of Interior from 2012 to 2025.	Vital status and date of death

CKD-DMIS, Chronic Kidney Disease–Disease Management Information System; HD, haemodialysis; HDC, Health Data Centre; ICD, International Statistical Classification of Diseases and Related Health Problems; KRT, kidney replacement therapy; MoPH, Ministry of Public Health; NHSO, National Health Security Office; PD, peritoneal dialysis; uACR, urine albumin-to-creatinine ratio; UCS, Universal Coverage Scheme; uPCR, urine protein-to-creatinine ratio.

The Universal Coverage Scheme (UCS) insures approximately 70% of the Thai population. The NHSO as the public payer oversees all UCS claims. The HDC captures laboratory data from all MoPH-affiliated and participating hospitals across the country, representing the majority of healthcare providers nationwide. Linkage across the HDC, CKD-DMIS, e-claim and Death Registry databases will be performed deterministically using the encrypted 13-digit Thai national identification number, allowing patients to be tracked across hospitals within the linked national system while minimising duplication and linkage error. Because KRT reimbursement and deaths are recorded in central national databases, these outcomes can still be ascertained even if patients move between hospitals within the system. For patients without a subsequent record of KRT initiation or death, follow-up will be censored at the date of their last recorded healthcare contact or claim in the available databases. Patients who migrate out of the Universal Coverage Scheme to another insurance scheme will be administratively censored at the date of their last recorded UCS claim.

### Risk prediction and outcome definitions

The four-variable KFRE for non-North American populations will be used to calculate the 2- and 5-year risk of KF.^10 11^ Details of the KFRE formula are provided in online supplemental appendix 2. The 2009 CKD-EPI creatinine equation^18^ will be mainly used for eGFR calculation to identify patients with CKD and as input for the KFRE. Sensitivity analyses will be conducted using alternative eGFR equations, including the 2021 CKD-EPI creatinine equation^19^ and the Thai eGFR equation,^20^ with full formulae presented in online supplemental appendix 3.

KF will be defined as the initiation of KRT—either haemodialysis or peritoneal dialysis—maintained for more than 90 days, or receipt of a kidney transplant. Unplanned dialysis will be defined as the initiation of KRT with haemodialysis as the first modality using a temporary, non-tunnelled central venous catheter. Hospitals will be categorised as primary care, which includes community hospitals at the district level, or secondary/tertiary care, which includes provincial hospitals, regional hospitals, university hospitals and specialised hospitals affiliated with the Department of Medical Services, MoPH. Comorbidities will be identified from International Statistical Classification of Diseases and Related Health Problems 10^th^ revision (ICD-10) codes recorded in the e-claim database, with the full code list provided in online supplemental table 1.

### Analysis plan

#### Descriptive analysis

We will conduct descriptive analyses to characterise CKD care within the Thai health system over the study period with cohort entry permitted from 1 January 2017 to 31 January 2025 and outcomes followed through 1 September 2025. Analysis will include examining the annual prevalence of CKD stages 3, 4 and 5; the availability of urine test results (uACR and uPCR); and patient characteristics including age, sex, diabetes, cardiovascular disease and hypertension. We will describe healthcare setting (primary vs secondary care), the proportion of patients initiating KRT, the proportion starting KRT in a planned or unplanned manner and trends in all-cause mortality. Additionally, we will compare patients managed in primary vs secondary/tertiary care, patterns of urine testing and the incidence of unplanned KRT and how these have evolved over time, particularly during the COVID-19 pandemic in Thailand (March 2020 to December 2021) and the implementation of national dialysis policy reform under the UCS implemented on 1 February 2022^21^ which shifted from a peritoneal-dialysis (PD) first policy to a free choice KRT. Furthermore, to assess potential selection bias driven by missing albuminuria data, we will extract baseline characteristics (eg, demographics, eGFR and comorbidities) of patients who met the eGFR inclusion criteria but were excluded solely due to missing uACR/uPCR, and statistically compare them against our included cohort.

#### External validation of the four-variable KFRE

We will perform an external validation of the four-variable KFRE to assess its predictive performance for the 2- and 5-year risk of KF in the study population. The primary outcome is the first initiation of KRT due to KF. Patients will be followed from the index date until the relevant censoring point, which will be 2 or 5 years after the index date, KRT initiation, death or 1 September 2025, whichever occurs first. Follow-up will be capped at 5 years to match the KFRE prediction horizon.

The primary analysis will evaluate the KFRE using a standard cause-specific hazard framework (death-censored approach) to align with the original KFRE derivation and multinational validation methodology.^10 11^ Calibration will be assessed comprehensively at the 2- and 5-year horizons without relying on arbitrary statistical thresholds,^22^ as a calibration slope of 1 does not independently guarantee good calibration. We will evaluate calibration-in-the-large (quantified by the observed-to-expected [O/E] ratio and the calibration intercept) and the calibration slope.^23^ Moderate calibration will be assessed visually across the entire range of predicted risks using smoothed, flexible calibration curves. To appropriately account for censoring when deriving these curves at fixed time horizons, the observed risks will be estimated using pseudo-observations from the Aalen-Johansen estimator.^23^

If miscalibration is detected, we will evaluate multiple recalibration strategies to identify the most efficient and parsimonious model. We will simultaneously derive three updated models: (1) recalibration-in-the-large (updating the baseline survival function only, estimated via post-estimation from the cohort’s data), (2) slope recalibration (adjusting both the baseline survival and the magnitude of the linear predictor) and (3) full model revision (re-estimating all individual predictor coefficients). The performance of these updated models will be compared against the original KFRE and each other using measures of discrimination, calibration (O/E ratio, calibration slope and flexible calibration curves) and overall fit (Brier score). To explicitly balance predictive improvement against model complexity, we will compare the models using information criteria such as the Akaike Information Criterion (AIC) or Bayesian Information Criterion (BIC), which apply a penalty for greater model complexity. The final chosen model will be the least complex updating method that achieves optimal calibration and clinical utility without overfitting. To correct for optimism during this model updating process, internal validation via bootstrapping will be performed on the full dataset to derive an optimism-adjusted shrinkage factor^23^ .

#### Sensitivity and subgroup analyses

Sensitivity and subgroup analyses will be conducted to assess the robustness and generalisability of the KFRE under varying assumptions and population characteristics.

First, subgroup analyses will be performed to evaluate model performance across distinct demographic and clinical strata. These will include stratifications by CKD stage, diabetes status, sex, age group (<65 versus ≥65 years) and baseline albuminuria category (A1: uACR <30 mg/g; A2: 30–300 mg/g; and A3: >300 mg/g, as defined by KDIGO).

Second, to assess the impact of predictor measurement and missing data, several analyses will be conducted. We will evaluate performance using alternative eGFR equations, specifically the 2021 CKD-EPI creatinine equation and the Thai eGFR equation. We will also compare performance between patients with directly measured uACR and those whose uACR was converted from a uPCR. To evaluate whether the 12-month measurement interval utilised in the primary analysis distorts baseline risk due to disease progression, a sensitivity analysis will restrict the cohort to patients whose albuminuria and eGFR were measured within a narrow ±90 day window. Furthermore, a multiple imputation analysis—incorporating auxiliary variables such as age, sex, eGFR, care settings and comorbidities—will be conducted to include the broader CKD population initially excluded due to missing uACR/uPCR data, explicitly testing whether this restriction artificially inflates apparent model performance.

Third, alternative outcome definitions and modelling frameworks will be explored. We will apply an alternative biological outcome definition for KF, defined as a non-rebounding eGFR<6 mL/min/1.73 m² persisting for more than 90 days or the initiation of chronic KRT. Additionally, to account for the potential overestimation of absolute risk when treating death as a censoring event, we will formally evaluate the competing risk of death using the Fine and Gray subdistribution hazard model.

Finally, a predefined temporal sensitivity analysis will investigate whether KFRE performance is altered by major health system disruptions. We will independently evaluate model performance across distinct periods to capture the effects of the COVID-19 pandemic (March 2020–December 2021) and the UCS dialysis policy reform implemented on 1 February 2022, which marked the transition from a ‘PD-First’ policy to a ‘Free-Choice’ treatment modality policy.^21^

#### Impact assessment of KFRE-guided referral compared with conventional criteria

We will evaluate the potential impact of implementing the KFRE in the referral system compared with both the conventional referral criteria recommended by the Thai CKD guideline 2022 and referral pattern observed in real-world practice. The guideline-based criteria include eGFR<30 mL/min/1.73 m², uACR >300 mg/g, uPCR>500 mg/g, an eGFR decline exceeding 5 mL/min/1.73 m²/year or a decline>25% from baseline.^24^

**Figure 1:**
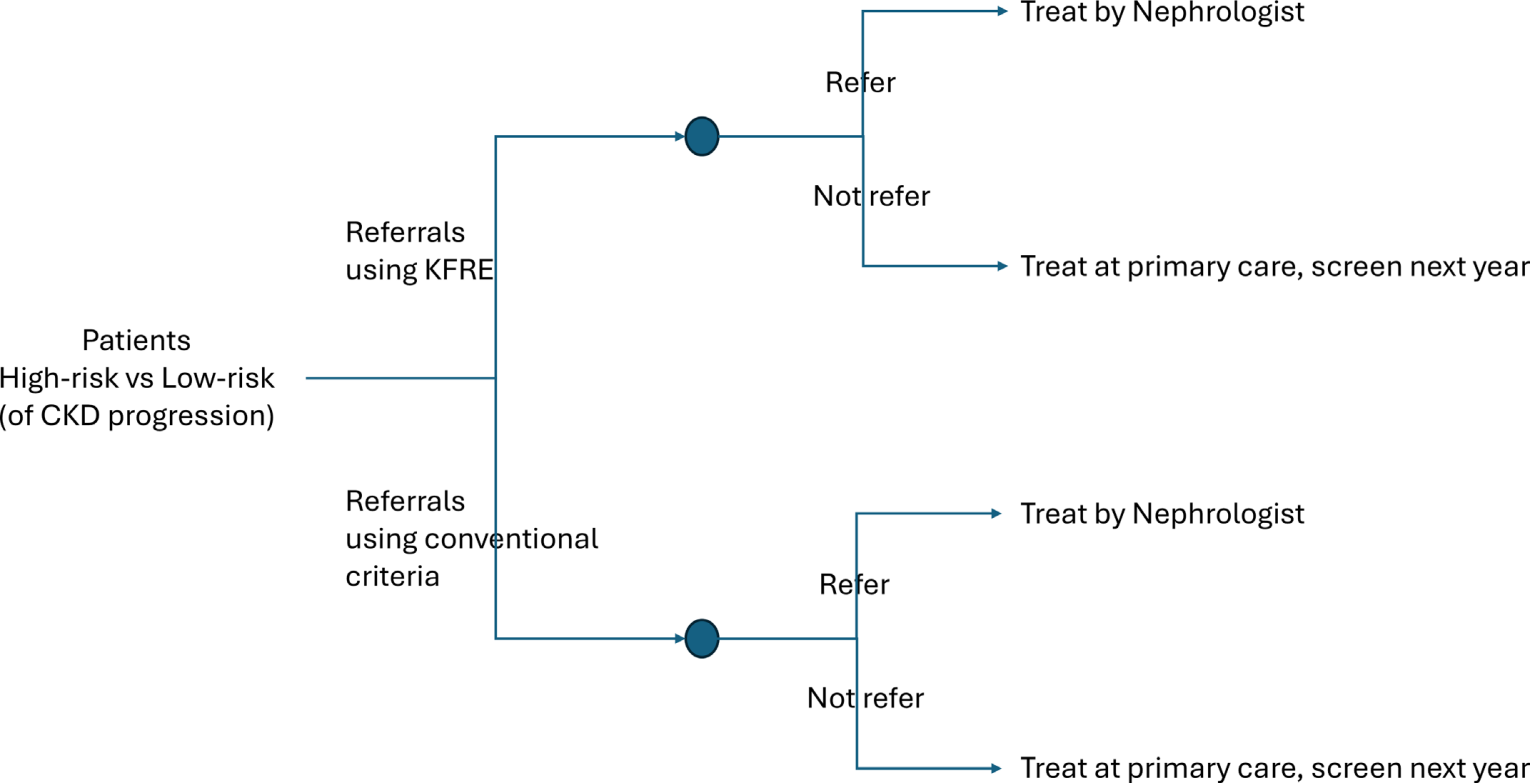
Choices of using different referral guidance. Patients were divided into six groups according to referral status by high KFRE vs conventional criteria and their 5-year outcomes: unplanned KRT, planned KRT or no KRT. Groups A–C represent additional referrals under KFRE, B–D represent patients referred under both criteria and E–F represent patients referred only by conventional criteria. CKD, chronic kidney disease; KFRE, kidney failure risk equation; KRT, kidney replacement therapy.

To compare referral strategies, a decision tree combined with a Markov model of CKD progression will be used to estimate the long-term outcomes under KFRE-guided referral vs conventional criteria and observed practice. The model structure is illustrated in figures1  2. Transition probabilities between CKD stages, mortality rates, probabilities of initiating dialysis and utility values associated with each health state will be incorporated, drawing on the datasets outlined in table 1.

**Figure 2:**
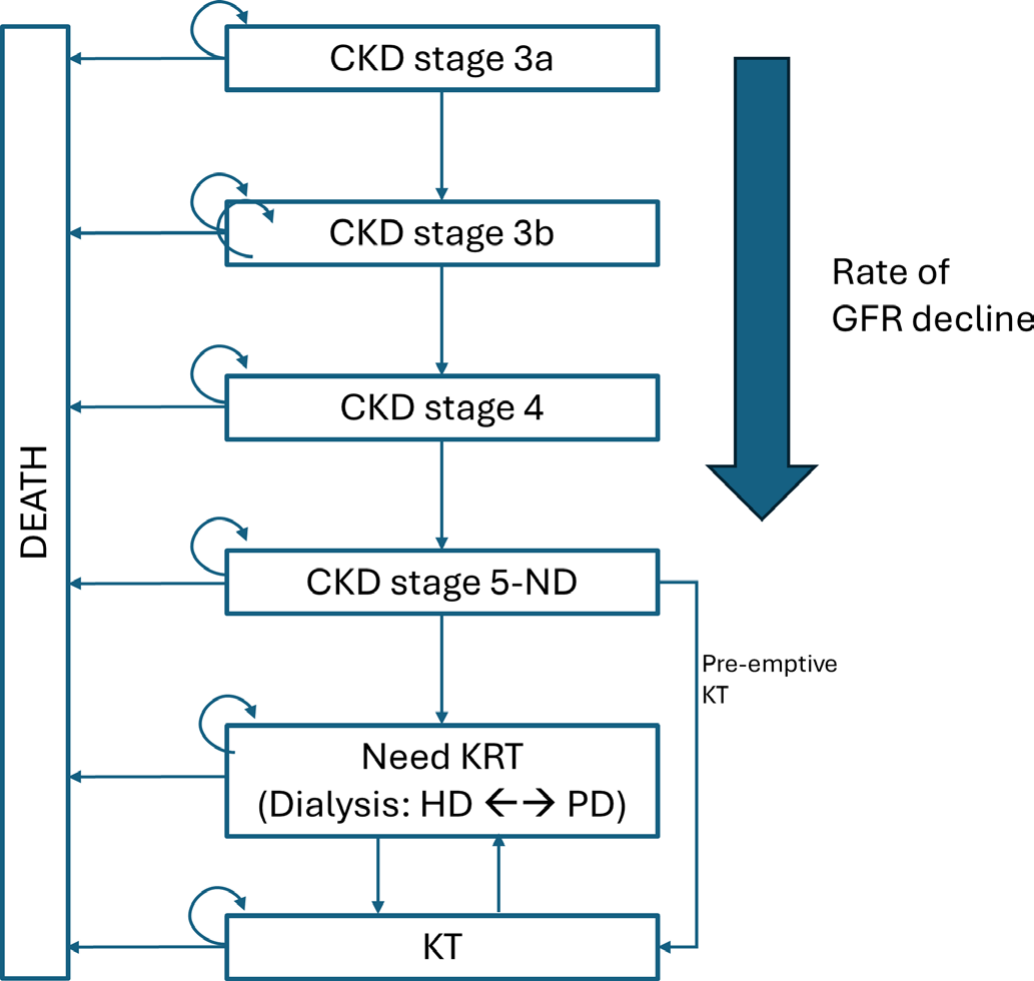
Markov model for CKD progression model structure representing transitions across CKD stages (3a to 5-ND; non-dialysis), initiation of kidney replacement therapy (KRT: HD, PD or pre-emptive KT) and death. Progression is determined by the rate of GFR decline, with transitions between dialysis modalities and to transplantation incorporated. CKD, chronic kidney disease; GFR, glomerular filtration rate; HD, haemodialysis; PD, peritoneal dialysis; KRT, kidney replacement therapy; KT, kidney transplant.

The impact assessment will address three key perspectives:

1. **Clinical perspective:** We will compare rates of unplanned dialysis initiation rates under KFRE-guided referral, guideline-based referral and observed practice, as indicators of timeliness and quality of referral. In addition, other patient-level outcomes will include eGFR slope as a marker of CKD progression, the proportion of patient initiating KRT in a planned manner and overall survival.
2. **Health system perspective:** We will assess how KFRE-guided referral affects the distribution of patients across primary, secondary/tertiary care settings compared with existing approaches. This will include quantifying reclassification (patients newly eligible or ineligible for referral) and examining the downstream implications for service delivery.
3. **Health economic perspective:** We will evaluate the economic impact of implementing KFRE-guided referral from both healthcare payer and societal perspectives. Costs will include outpatient nephrology consultations, laboratory testing, dialysis initiation and long-term treatment. The societal perspective will additionally account for non-medical expenditure, productivity losses and informal care, using the standard cost list for health technology assessment in Thailand.^25^

**Figure 3:**
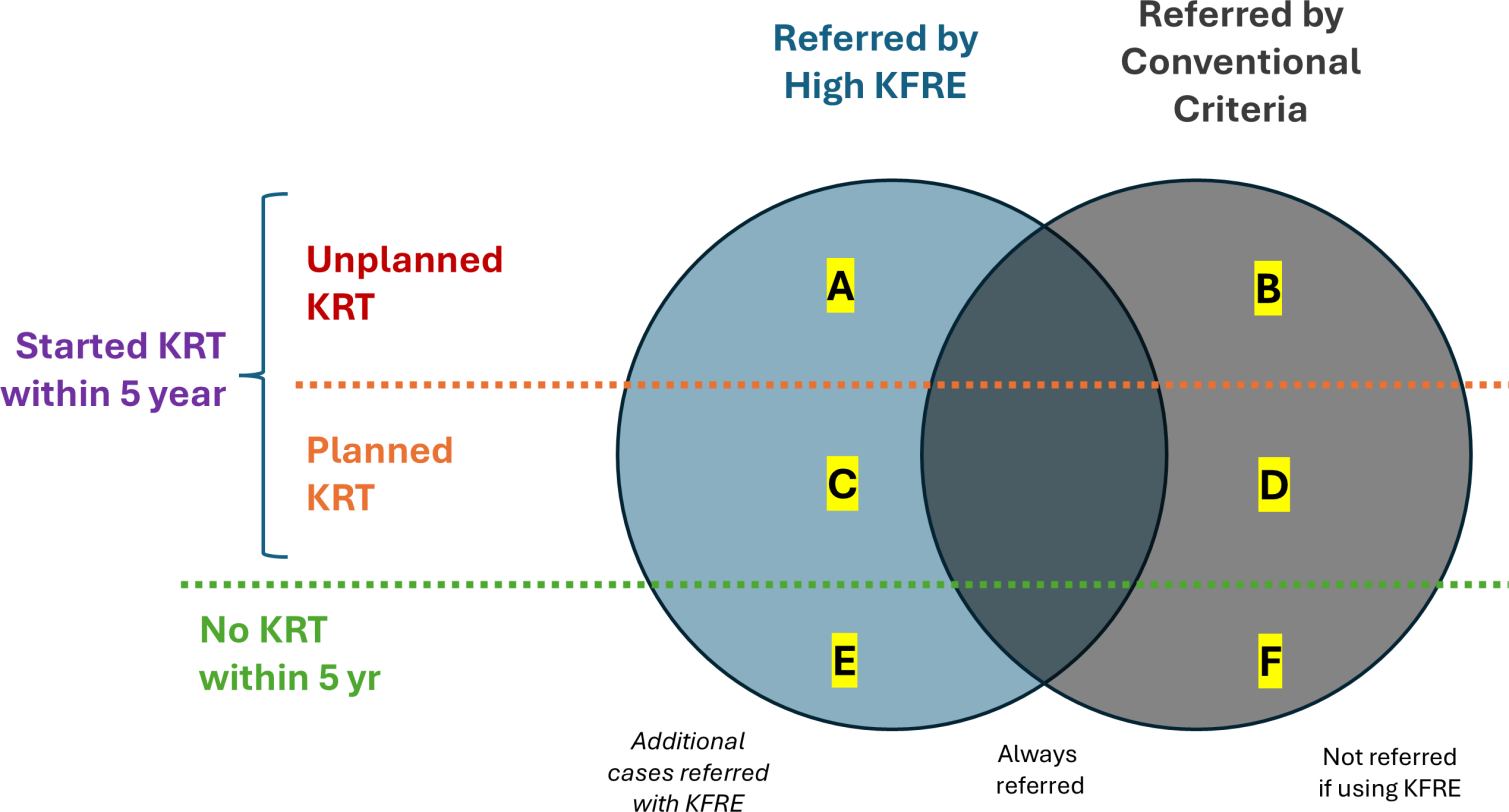
Patients’ classification based on referral criteria and outcomes. Patients were divided into six groups according to referral status by high KFRE vs conventional criteria and their 5 year outcomes: unplanned KRT, planned KRT or no KRT. Groups A–C represent additional referrals under KFRE, B–D represent patients referred under both criteria and E–F represent patients referred only by conventional criteria. KFRE, kidney failure risk equation; KRT, kidney replacement therapy.

Patients will be categorised into six mutually exclusive groups based on their referral status and clinical outcomes over 5 years (figure 3). Outcomes include planned or unplanned KRT initiation and survival without KRT.

#### Management of missing data

Missing data will be reported with count and percentage. A complete case approach will be utilised for the primary analysis. To quantify potential selection bias, the baseline characteristics of patients excluded solely due to missing predictor data (eg, missing uACR or uPCR measurements) will be extracted and statistically compared with the included cohort. Furthermore, to evaluate model performance under alternative missingness assumptions, a sensitivity analysis will be conducted using multiple imputation by chained equations. This imputed analysis will also be evaluated using the standard cause-specific hazard framework. To ensure the imputation model is congenial with the survival framework, it will explicitly incorporate the event indicators and the Nelson–Aalen estimators of the cumulative baseline hazards for both KF and death.

#### Software used and reporting

Data manipulation will be performed using SQL and R, and statistical analyses will be conducted using R and Stata/SE version 19. All analyses and reporting of the model validation and re-calibration section will follow the TRIPOD (Transparent Reporting of a multivariable prediction model for Individual Prognosis or Diagnosis) guideline^26^ .

### Sample size and power calculations

As this is a retrospective study using secondary data, all available records will be included to maximise generalisability, which is feasible as the dataset contains over 5000 KF events. Following the simulation-based approach,^27^ parameters for the linear predictor distribution and event and censoring probabilities were derived from the original KFRE development cohort.^10^ Simulations (1000 iterations) indicated the need for approximately 920 events at 5 years to achieve a target SE of 0.051 for the calibration slope of the 5-year KFRE and 0.095 for the 2-year KFRE. A power calculation using the same simulation-based method will also be conducted on the study dataset to obtain empirical estimates.

### Patient and public involvement

Patients and the public were not involved in the design, conduct, reporting or dissemination plans of this study. However, the research team will engage relevant stakeholders—including policymakers, nephrologists, internists, general practitioners, patient representatives and agencies such as the MoPH and the NHSO—through structured consultations to support interpretation of the findings and guide dissemination strategies, particularly for implementing risk-based referral approaches in practice.

### Study timeline

Data extraction and preparation began in January 2026. Model validation and impact analyses are planned between April and August 2026.

## Ethics and dissemination

This study uses de-identified secondary data from national health databases, ensuring that individuals cannot be directly identified. Ethical approval was obtained from the Institute for the Development of Human Research Protections, Thailand (COA No. IHRP2025110), the Imperial College Research Ethics Committee (ref no. 7837328) and the London School of Hygiene and Tropical Medicine Research Ethics Committee (ref no. 32713). As the data were anonymised prior to analysis and posed minimal risk, the requirement for informed consent was waived. Data access was granted by the NHSO under a formal data use agreement, and all analyses will be conducted within a secure, access-controlled environment in compliance with national data protection standards.

Study findings will be disseminated through peer-reviewed publications, national and international conferences and targeted policy briefs. Stakeholder engagement, including with policymakers, clinicians and patient representatives, will support interpretation of results and inform dissemination strategies to facilitate translation into practice.

## Supplementary material

10.1136/bmjopen-2025-114382 online supplemental file 1

10.1136/bmjopen-2025-114382 online supplemental file 2
