# Supplementary material for: Validation of the kidney failure risk equation and its impact on referral strategies for chronic kidney disease: protocol for a retrospective cohort study using national claims and laboratory data in Thailand
Source: BMJ Open. 2026 May 15;16(5):e114382. doi: 10.1136/bmjopen-2025-114382 (PMC13182311; doi:10.1136/bmjopen-2025-114382)
Supplement: online supplemental file 2 [file bmjopen-16-5-s002.docx]

## Supplemental appendix 1. Eligibility criteria

Inclusion criteria

1. Adults aged ≥18 years.
2. At least two recorded serum creatinine values with eGFR <60 ml/min/1.73m², measured ≥90 days apart, with no intermediate eGFR ≥60 ml/min/1.73m² (CKD stage 3–5).
3. Availability of a recorded quantitative urine albumin-to-creatinine ratio (UACR) within ±12 months of the index date. If UACR is not available, a urine protein-to-creatinine ratio (UPCR) recorded in the same period will be used and converted to UACR using validated equation.

Exclusion criteria

1. Evidence of chronic dialysis for >3 months prior to the index date.
2. Kidney transplantation prior to the index date.
3. Recorded as receiving palliative care prior to or at the index date.
4. Main health insurance scheme other than the Universal Coverage Scheme at the latest follow-up date, to ensure complete capture of outcome events in the National Health Security Office (NHSO) database.

## Supplemental appendix 2. The 4-variable kidney failure risk equation (KFRE) for non-North American

The 4-variable KFRE for non-North American equation:

***5-year ESKD risk =* 1 - 0.9365^exp(βsum)^**

***2-year ESKD risk: =* 1 - 0.9832 ^exp(βsum)^**

*where* βsum = -0.2201 × (age/10 – 7.036)
 + 0.2467 × (male – 0.5642)
 – 0.5567 × (eGFR/5 – 7.222)
 + 0.4510 × (logACR – 5.137)

## Supplemental appendix 3. Equations for estimated glomerular filtration rate (eGFR) using serum creatinine (SCr)

2009 CKD-EPI Creatinine equation (for non-black population)^(1)^

eGFR = A x (SCr/B)^C^ x 0.993^age,^

*where A, B, and C are the following:*

|  |  | **A** | **B** | **C** |
| --- | --- | --- | --- | --- |
| **Female** | **SCr ≤ 0.7** | 144 | 0.7 | -0.329 |
|  | **SCr > 0.7** | 144 | 0.7 | -1.209 |
| **Male** | **SCr ≤ 0.9** | 141 | 0.9 | -0.411 |
|  | **SCr > 0.9** | 141 | 0.9 | -1.209 |

2021 CKD-EPI Creatinine equation^(2)^

eGFR = 142 x (SCr/A)^B^ x 0.9938^age,^

*where A and B are the following:*

|  |  | **A** | **B** |
| --- | --- | --- | --- |
| **Female** | **SCr ≤ 0.7** | **0.7** | **-0.241** |
|  | **SCr > 0.7** | **0.7** | **-1.2** |
| **Male** | **SCr ≤ 0.9** | **0.9** | **-0.302** |
|  | **SCr > 0.9** | **0.9** | **-1.2** |

Thai eGFR creatinine equation^(3)^

eGFR = 375.5 x SCr^(-0.848)^ x Age ^(-0.364)^ x 0.712 (if female)

1.Levey AS, Stevens LA, Schmid CH, Zhang YL, Castro AF, 3rd, Feldman HI, et al. A new equation to estimate glomerular filtration rate. Ann Intern Med 2009;150:604-12.

2.Inker LA, Eneanya ND, Coresh J, Tighiouart H, Wang D, Sang Y, et al. New Creatinine- and Cystatin C-Based Equations to Estimate GFR without Race. N Engl J Med 2021;385:1737-49.

3.Praditpornsilpa K, Townamchai N, Chaiwatanarat T, Tiranathanagul K, Katawatin P, Susantitaphong P, et al. The need for robust validation for MDRD-based glomerular filtration rate estimation in various CKD populations. Nephrol Dial Transplant 2011;26:2780-5.

**Supplemental Table 1: ICD-10 codes for comorbidities**

| **Comorbidity** | **ICD-10 codes** |
| --- | --- |
| Myocardial infarction | I21-I22, I252 |
| Chronic ischemic heart disease | I25 |
| Congestive heart failure | I099,I110,I255,I130,I132,I42,I43,I50,P290 |
| Peripheral vascular disease | I70,I71,I731,I738,I739,I771.I790,I792,K551,K558,K559,Z958,Z959 |
| Cerebrovascular disease | G45,G46,I60-I69,H340 |
| Dementia | F01-F03,F051,G30,G311 |
| Hemiplegia/paraplegia | G041,G114,G801,G802,G81,G82,G830-4,G839 |
| Chronic lung disease | I278,I279,J40-7,J60-7,J684,J701,J703 |
| Rheumatologic disease | M05,M06,M315,M32,M33,M34,M351,M353,M360 |
| Peptic ulcer disease | K25-28 |
| Diabetes mellitus without complications | E100,E101,E106,E108,E109,E110,E111,E116,E118,E119,E120,E121,E126, E128,E129,E130,E131,E136,E138,E139,E140,E141,E146,E148,E149 |
| Diabetes mellitus with chronic complications | E102-E105,E107,E112,E115,E117,E122-E125,E127,E132-E135,E137,E142-E145,E147 |
| Mild liver disease | B18,K700-K703. K709,K713-K715,K717,K73,K74,K760,K762-K764,K768,K769.Z944 |
| Moderate/severe liver disease | I850,I856,I864,I982,K704,K711,K721,K729,K765-K767 |
| Any malignancy | C00-C26,C30-C34.C37-C41.C43,C45-C58,C60-C76,C81-C85,C88,C90-C97 |
| Metastatic solid tumour | C77-C80 |
| HIV/AIDS | B20-B22 (AIDS), B24 (asymptomatic HIV) |
| Hypertension | I10-15 |
| Acute kidney injury | N17 |
| Chronic kidney disease | N181-N185 |
| Palliative care encounter | Z515+ procedure code 71.8 |
