# Supplementary material for: Validation of the kidney failure risk equation and its impact on referral strategies for chronic kidney disease: protocol for a retrospective cohort study using national claims and laboratory data in Thailand
Source: BMJ Open. 2026 May 15;16(5):e114382. doi: 10.1136/bmjopen-2025-114382 (PMC13182311; doi:10.1136/bmjopen-2025-114382)
Supplement: Supplementary file 3 [file bmjopen-2025-114382.reviewer_comments.pdf]

# PEER REVIEW HISTORY

BMJ Open publishes all reviews undertaken for accepted manuscripts. Reviewers are asked to complete a checklist review form and are provided with free text boxes to elaborate on their assessment. These free text comments are reproduced below.

## ARTICLE DETAILS

### Title (Provisional)

Validation of the Kidney Failure Risk Equation and its impact on referral strategies for chronic kidney disease: protocol for a retrospective cohort study using national claims and laboratory data in Thailand

### Authors

Phannajit, Jeerath; Narkpaichit, Chirod; Angkurawaranon, Chaisiri; Aramrat, Chanchanok; Cleary, Faye; Major, Rupert; Pichaiwong, Warangkana; Anutrakulchai, Sirirat; Praditpornsilpa, Kearkiat; Turner, Hugo C; Nitsch, Dorothea

---

## VERSION 1 - REVIEW

---

| | |
|---|---|
| **Reviewer** | **1** |
| **Name** | **Kaewput, Wisit** |
| **Affiliation** | **Phramongkutklao College of Medicine** |
| **Date** | **22-Jan-2026** |
| **COI** | **None** |

---

This is an interesting manuscript. however some minor concern should be clarified.

Minor concern include:

1. The cohort is restricted to individuals with recorded uACR (or uPCR convertible to uACR) within ±12 months, which in routine practice is often missing and is not missing at random (patients tested tend to be higher risk or under better-resourced care). This restriction may generate a selected cohort that is systematically different from the broader CKD stage 3–5 population, and will likely inflate apparent performance and distort calibration.

2. The protocol proposes no imputation and only "available data" analysis; this is not appropriate when the principal predictor (albuminuria) is a major driver of missingness and selection.

3. CKD stage 3–5 defined by two eGFR <60 ≥90 days apart is standard, but the index date as the second qualifying eGFR may introduce bias in a dynamic healthcare setting: patients must survive and remain under observation long enough to obtain the second test, and testing frequency differs by risk and setting.

4. Allowing uACR/uPCR within ±12 months of index date is very wide. In a progressive disease, albuminuria and eGFR can change substantially, so predictor measurement may not reflect the risk state at baseline.

5. Kidney failure is defined primarily as initiation of KRT maintained >90 days or transplant. This is a health-system-dependent endpoint (access, policy, modality availability), especially in Thailand where policy reforms (PD-first to free choice) and capacity constraints influence "starting dialysis." Although you propose an alternative definition using sustained eGFR <6, the primary endpoint remains treatment initiation, which may vary across regions, hospitals, and over time.

6. "Miscalibration" thresholds (slope 0.90–1.10; intercept ±0.10) are stated but not justified; calibration assessment should be more nuanced and include calibration-in-the-large, flexible calibration curves, and time-to-event calibration at 2 and 5 years.

7. Recalibration is described as "adjusting baseline survival function using Cox models," but details are insufficient: Will you perform recalibration-in-the-large only, slope recalibration, or full recalibration?

8. How will you estimate baseline survival at fixed horizons (2 and 5 years) under censoring?

9. How will you avoid optimism in the recalibration step (internal validation within development period)?

10. A simple temporal split is reasonable, but choosing validation as "index dates on or after 1 Jan 2019" appears arbitrary. Also, policy changes in 2022 may make "post-2019" heterogeneous.

11. You plan Fine-Gray only as a sensitivity analysis, yet death is common in CKD stages 3-5 and directly affects observed incidence of KRT. For risk prediction of KRT initiation, the estimand should be clearly defined: cause-specific vs subdistribution risk at 2/5 years. Clear statement of the estimand and primary modelling framework for time-to-event prediction with competing risks; at minimum, parallel reporting using cause-specific and subdistribution approaches, with interpretation focused on the decision context (referral).

12. Complete-case analysis is likely to bias both validation and impact modelling, especially when missingness is systematic (uACR/uPCR, creatinine testing frequency, care setting). Multiple imputation (with appropriate survival framework), or a principled missingness strategy; at minimum, a plan to quantify and mitigate selection bias and to report how conclusions change under alternative missingness assumptions.

13. Provide more detail on governance and data linkage quality (linkage error, duplicates, migration between schemes/hospitals).

---

## VERSION 1 - AUTHOR RESPONSE

Reviewer#1's comment

1. The cohort is restricted to individuals with recorded uACR (or uPCR convertible to uACR) within ±12 months, which in routine practice is often missing and is not missing at random (patients tested tend to be higher risk or under better-resourced care). This restriction may generate a selected cohort that is systematically different from the broader CKD stage 3–5 population, and will likely inflate apparent performance and distort calibration.

2. The protocol proposes no imputation and only "available data" analysis; this is not appropriate when the principal predictor (albuminuria) is a major driver of missingness and selection.

*Authors' response (to comments 1&2): We thank the reviewer for these important methodological comments. We agree that missing albuminuria data in routine practice are unlikely to be missing at random, as testing is related to patient risk and care setting, and that reliance on a complete-case analysis alone may introduce selection bias and distort apparent model performance. We have therefore revised the protocol accordingly. First, we will compare the baseline clinical and demographic characteristics of the included cohort with those of patients excluded solely because of missing uACR/uPCR data to assess potential selection bias* ***(p 10-11)****. Second, we have added multiple imputation by chained equations as a prespecified sensitivity analysis to examine the robustness of KFRE performance and calibration under alternative missing-data assumptions* ***(p12 & 14)****.*

3. CKD stage 3–5 defined by two eGFR <60 ≥90 days apart is standard, but the index date as the second qualifying eGFR may introduce bias in a dynamic healthcare setting: patients must survive and remain under observation long enough to obtain the second test, and testing frequency differs by risk and setting.

***Authors' response:*** *We agree that defining the index date as the second qualifying eGFR may introduce bias, as patients must survive and remain under observation long enough to undergo repeat testing, and testing frequency may vary by clinical risk and care setting. However, because our primary aim is to validate the KFRE in patients with confirmed CKD, we have retained the requirement for two eGFR measurements <60 mL/min/1.73 m² at least 90 days apart, consistent with KDIGO definitions and to reduce misclassification of acute kidney injury. To address the potential bias while preserving this CKD definition, we have revised the protocol so that the index date (time zero) is defined as the date of the albuminuria measurement (uACR or uPCR),* ***(p.7)*** *following the approach used in a previous UK primary care KFRE validation study. (Major et al. PLOS Medicine 2019;16:e1002955)*

4. Allowing uACR/uPCR within ±12 months of index date is very wide. In a progressive disease, albuminuria and eGFR can change substantially, so predictor measurement may not reflect the risk state at baseline.

*Author response: We agree that, in a progressive disease, a 12-month interval between eGFR and albuminuria measurements may not perfectly reflect a single baseline risk state. However, in routine care, requiring near-contemporaneous measurements in the primary analysis would substantially reduce sample size and may not reflect real-world testing patterns. We have therefore added a prespecified sensitivity analysis restricting the cohort to patients with eGFR and albuminuria measured within a ±90-day window.* ***(p.12)***

5. Kidney failure is defined primarily as initiation of KRT maintained >90 days or transplant. This is a health-system-dependent endpoint (access, policy, modality availability), especially in Thailand where policy reforms (PD-first to free choice) and capacity constraints influence “starting dialysis.” Although you propose an alternative definition using sustained eGFR <6, the primary endpoint remains treatment initiation, which may vary across regions, hospitals, and over time.

*Author response: We agree that KRT initiation is a health-system-dependent endpoint that may vary with access, service capacity and policy over time. We have therefore revised the protocol in three ways. First, we now explicitly acknowledge this limitation in the Strengths and limitations of this study section* ***(p.4)****. Second, we have added a predefined temporal sensitivity analysis to evaluate whether KFRE performance differs during major health-system changes, including the COVID-19 period and the February 2022 UCS dialysis policy reform. Third, we retain a sensitivity analysis using an alternative biological definition of kidney failure based on sustained eGFR <6 mL/min/1.73 $m^2$ or chronic KRT initiation* ***(p13)****.*

6. “Miscalibration” thresholds (slope 0.90–1.10; intercept ±0.10) are stated but not justified; calibration assessment should be more nuanced and include calibration-in-the-large, flexible calibration curves, and time-to-event calibration at 2 and 5 years.

*Author response: We thank the reviewer for this important methodological point and agree that rigid thresholds for miscalibration are arbitrary. We have therefore removed the prespecified slope and intercept thresholds from the protocol. Calibration will now be assessed more comprehensively using calibration-in-the-large (O/E ratio and calibration intercept), calibration slope, and flexible smoothed calibration curves at the 2- and 5-year horizons* ***(p.11)****.*

7. Recalibration is described as "adjusting baseline survival function using Cox models," but details are insufficient: Will you perform recalibration-in-the-large only, slope recalibration, or full recalibration?

*Author response: We agree that the previous description lacked sufficient detail. The revised protocol now specifies that, if miscalibration is identified, we will evaluate three updating strategies: recalibration-in-the-large, slope recalibration, and full model revision. These approaches will be compared using measures of calibration, overall fit and model parsimony to select the least complex approach that provides adequate performance* ***(p11-12)***.

8. How will you estimate baseline survival at fixed horizons (2 and 5 years) under censoring?

*Author response: We have clarified this in the revised protocol. Observed risks at the 2- and 5-year horizons will be estimated using pseudo-observations derived from the Aalen-Johansen estimator, allowing calibration to be assessed appropriately in the presence of censoring* ***(p.11)***.

9. How will you avoid optimism in the recalibration step (internal validation within development period)?

*Author response: We have revised the protocol to specify internal validation by bootstrapping during the model updating process. This will be used to obtain optimism-adjusted performance estimates and derive a shrinkage factor to reduce overfitting in the recalibrated model* ***(p.9)***.

10. A simple temporal split is reasonable, but choosing validation as "index dates on or after 1 Jan 2019" appears arbitrary. Also, policy changes in 2022 may make "post-2019" heterogeneous.

*Author response: We thank the reviewer for this comment and agree that a 1 January 2019 split is arbitrary and that the 2022 Universal Coverage Scheme policy reform may introduce temporal heterogeneity. We have therefore abandoned the temporal split and will use the full eligible national dataset for recalibration, with bootstrap internal validation to account for optimism. In addition, we have added a predefined temporal sensitivity analysis comparing KFRE performance and calibration before and after the February 2022 policy reform to assess potential calibration drift associated with this major health-system change* ***(p.10)***.

11. You plan Fine-Gray only as a sensitivity analysis, yet death is common in CKD stages 3-5 and directly affects observed incidence of KRT. For risk prediction of KRT initiation, the estimand should be clearly defined: cause-specific vs subdistribution risk at 2/5 years. Clear statement of the estimand and primary modelling framework for time-to-event prediction with competing risks; at minimum, parallel reporting using cause-specific and subdistribution approaches, with interpretation focused on the decision context (referral).

*Author response: We agree that death is a common competing event in CKD stages 3–5 and that the estimand should be clearly defined. We have therefore clarified in the revised protocol that the primary analysis will use a cause-specific hazard framework, consistent with the original KFRE derivation and most external validations. To address the effect of competing mortality on absolute risk, we have also specified a sensitivity analysis using the Fine and Gray subdistribution hazard model at the 2- and 5-year horizons* ***(p.11).***

12. Complete-case analysis is likely to bias both validation and impact modelling, especially when missingness is systematic (uACR/uPCR, creatinine testing frequency, care setting). Multiple imputation (with appropriate survival framework), or a principled missingness strategy; at minimum, a plan to quantify and mitigate selection bias and to report how conclusions change under alternative missingness assumptions.

*Author response: We agree that complete-case analysis may introduce selection bias when missingness is systematic. We have therefore revised the protocol to include two measures. First, we will compare the baseline characteristics of included patients with those excluded solely because of missing predictor data to quantify potential selection bias. Second, we will perform a prespecified multiple imputation sensitivity analysis within an appropriate survival framework, incorporating event indicators and the Nelson–Aalen cumulative hazard estimators for kidney failure and death, to assess the robustness of the findings under alternative missing-data assumptions.* ***(p.10-12 and 14)***

13. Provide more detail on governance and data linkage quality (linkage error, duplicates, migration between schemes/hospitals).

*Author response: We thank the reviewer for this helpful suggestion. We have revised the Data sources and population coverage section to provide further detail on data governance, linkage procedures and follow-up. Specifically, we clarify that the four national databases will be linked deterministically using the encrypted 13-digit national identification number, which minimises linkage error and duplication and allows patients to be tracked across hospitals within the linked national system. This includes ascertainment of centrally recorded outcomes such as KRT reimbursement and death, even if patients move between hospitals. For patients with no subsequent record of KRT initiation or death, follow-up will be censored at*

*the date of their last recorded healthcare contact within the available databases. We have also clarified how migration out of the Universal Coverage Scheme will be handled through administrative censoring at the date of the last recorded UCS claim.* ***(p.7-8)***.

---

We again thank the reviewer for the helpful comments and hope that the revisions have addressed the concerns satisfactorily.
